# Chromosome level genome assembly and annotation of *Hanseniaspora mollemarum* CBS 18055 strain

**DOI:** 10.1128/mra.01005-24

**Published:** 2025-01-29

**Authors:** Nicole Xanthe Bennis, Harshitha Indudhar Hanji, Marcel van den Broek, Jean-Marc Daran

**Affiliations:** 1 Department of Biotechnology, Delft University of Technology, Delft, the Netherlands; University of California Riverside, Riverside, California, USA

**Keywords:** yeasts, saccharomycotina, *Hanseniaspora*, genomes

## Abstract

Hanseniaspora species gained attention due to the ability of these species to ferment simple sugars and to actively contribute to the development of bouquet aromas in wine and cider fermentations. We present a chromosome-level assembly of an isolate of *Hanseniaspora mollemarum* that would enhance its potential applications.

## ANNOUNCEMENT

The *Hanseniaspora* genus comprises over 20 species including the recently described *Hanseniaspora gamundiae* (1), *Hanseniaspora smithiae* (2), *Hanseniaspora terricola* (3), and *Hanseniaspora mollemarum*. The latter species was isolated in a soil sample from a Dutch garden (CBS 15034) (4). Subsequently, new strains from Great-Britain and Hungary have been deposited at the Westerdijk Fungal Biodiversity Institute (https://wi.knaw.nl/). However, the sole *H. mollemarun* genome currently available derives from the CBS 15034 strain. To match the interest of the beverage industry for *Hanseniaspora* strains, it is critical to gain both physiological and genomic knowledge. Here, we report the genome sequence of a new strain of *H. mollemarum*.

The *H. mollemarum* CBS 18055 was isolated from an apple tree (*Malus domestica*) located in the south of France (43°01’24.7”N, 0°58’30.4”E). The sample was grown at 4°C on YPD medium (10 g L^−1^ yeast extract, 20 g L^−1^ Bacto-peptone, and 20 g L^−1^ glucose) supplemented with 3% ethanol. Single colonies were obtained by re-streaking the culture on a solid YPD medium supplemented with 25 µg mL^−1^chloramphenicol and 50 µg mL^−1^ ampicillin incubated at room temperature. This process was repeated thrice. Final isolates were stored in 30% (vol/vol) glycerol at −80°C.

Sanger sequencing of the ITS and the D1/D2 region of the rDNA of CBS 18055 amplified with primers ITS1/ITS4 (5) and NL1/NL4 (6), respectively, revealed a 99.98 and 100% identity to that of *H. mollemarum* CBS 15034, respectively.

For genome sequencing, total DNA was extracted using the Genomic-tip 100 /G kit protocol (Qiagen, Hilden, Germany). Shotgun library was prepared using Oxford_Nanopore Technologies' SQK-LSK109 kit (ONT, Oxford, United Kingdom) and sequenced on a MinION MK1B device with R10 flow cell. Raw FAST5 signal files were base-called using GPU Guppy (ONT, version 4.5.4) in high-accuracy mode. After filtering for length (>1 kb), 94,060 reads were obtained, yielding 1.04 Gb (N50: 19,089 bp), representing ~112-fold coverage of a *H. mollemarum* genome. Canu version 2.0 with settings genomeSize = 12 m, useGrid = 0, and nanopore-raw was used for *de novo* assembly yielding eight contigs (7). To correct errors in the assembly, a 150 bp read length TruSeq PCR-Free Illumina library (Illumina, San Diego, CA) with a 350 bp insert-size was sequenced on a NovaSeq 6000 (Illumina) by Novogene (Cambridge, United Kingdom) yielding 18,811,684 reads for a total of 2.82 Gigabases (307-fold coverage). Reads quality was assessed with FastQC v0.11.5. The genome was polished by mapping the untrimmed Illumina reads with Burrows-Wheeler Aligner (BWA version 0.7.15-r1142-dirty; default parameters used) (8, 9) to the assembly and further processed with SAMtools (version 1.3.1) (10, 11) and polished once by Pilon (version 1.18) with settings fix all (12).

This yielded a genome assembly of 9.18 Mb (N50 = 1.25 Mb) with a GC content of 34.96%, comprising eight contigs. Contig 1–7 (CHR2-to-8) showed telomeric sequences at both ends, whereas contig 8 (CHR1) had telomere sequences on one chromosome arm indicating that this contig also represented (part of) a linear chromosome (Table 1). Due to Canu’s being notoriously known to have difficulty in assembling circular contigs, the fastq files were assembled using UniCycler Hybrid assembler (13). A circular contig of 32.9 kbp was identified as mitochondrial chromosome (Table 1). Annotation of the polished assembly was performed using Funannotate v1.8.15 ((https://github.com/nextgenusfs/funannotate) (14) using ab initio gene predictors SNAP (v2013_11_29), Augustus (3.5.0), Genemark.HMM (ES Suite v4), and glimmerhmm (v3.0.4) in combination with default Funannotate databases, for example, pfam, interpro, andUniprot as previously described in (15).

**TABLE 1:** Statistics of the *H. mollemarum* assembly GCA_042466605.1

			Canu (ONT)	UniCycler hybrid
Assembly size (Mb)			9.18 (*n* = 105)	
N50 (Mb)			1.25 (*n* = 4)	
N90 (Kb)			105.20 (*n* = 9)	
	GenBank	Chr	Size (bp)	Size (bp)
contig1 (bp)	CP157652.1	Chr 8	1,960,557	
contig2 (bp)	CP157651.1	Chr 7	1,481,168	
contig3 (bp)	CP157650.1	Chr 6	1,331,897	
contig4 (bp)	CP157649.1	Chr 5	1,256,963	
contig5 (bp)	CP157648.1	Chr 4	1,027,777	
contig6 (bp)	CP157647.1	Chr 3	994,132	
contig7 (bp)	CP157646.1	Chr 2	662,091	
contig8 (bp)	CP157645.1	Chr 1	469,443	
contig9 – circular (bp)	CP157653.1	mtDNA		32,902
		Nuclear DNA (bp)	9,184,028	
		Total (bp)		9,216,930

## Data Availability

The genome reported in this publication was deposited at NCBI (https://www.ncbi.nlm.nih.gov/) under the BioProject accession number PRJNA977567. The BioProject includes the assembly GCA_042466605.1 and the associated Illumina and Nanopore reads SRX23808396 and SRX23808395, respectively. The annotation was deposited at the 4TU repository (https://data.4tu.nl/) under URL https://doi.org/10.4121/06bb6616-2da3-4211-8694-ff5ae37935dc.v1.
